# Using the gravitational energy of water to generate power by separation of charge at interfaces†
†Electronic supplementary information (ESI) available. See DOI: 10.1039/c5sc00473j



**DOI:** 10.1039/c5sc00473j

**Published:** 2015-03-26

**Authors:** Yajuan Sun, Xu Huang, Siowling Soh

**Affiliations:** a Department of Chemical and Biomolecular Engineering , National University of Singapore , 4 Engineering Drive 4 , Singapore 117585 , Singapore . Email: chessl@nus.edu.sg

## Abstract

When a fluid comes into contact with a solid surface, charge separates at the interface. This study describes a method that harvests the gravitational energy of water—available in abundance naturally, such as in rain and rivers—through the separation of charge at the interface. Essentially, it is found that water can be charged by flowing it across a solid surface under its own weight; thus, a continuous flow of water can produce a constant supply of power. After optimizing the system, a power of up to ∼170 μW (per Teflon tube of 2 mm in diameter) can be generated. The efficiency, defined as the energy generated by the system over the gravitational energy that the water losses, can reach up to ∼3–4%. In order to generate a continuous stream of positively-charged water, there should also be a constant production of negatively-charged species in the system. Experimental results suggest that the negative charge transfers constantly to the atmosphere due to dielectric breakdown of air. With regards to applications related to high electrical potential of water droplets, the amount of charge generated in a single water droplet is found to be equivalent to that produced by charging the water droplet with a high-voltage power supply operated at ∼5 kV. In general, the energy generated is clean, renewable, and technically simple and inexpensive to produce.

## Abstract 2

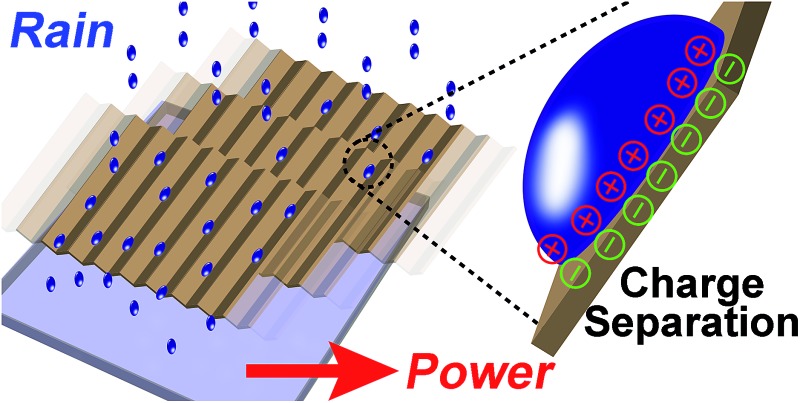
When water droplets (*e.g.*, from rain) flow down a solid surface due to gravity, they can generate power.

## Introduction

The world needs technologies that can generate clean and renewable energy, and especially those that are inexpensive to operate. One proven source of clean and renewable energy is the gravitational energy of water; it is known to produce a large amount of energy, for example, in the form of hydroelectricity. This method of harvesting the gravitational energy of water, however, is not without limitations, and has a few disadvantages.^1^

Energy can also be harvested by contact electrification—the phenomenon in which charge separates at the interface when two materials are brought into contact and are then separated. When the contact involves two solid surfaces, harvesting energy using this method has been challenging. Although the phenomenon has been observed for centuries, the mechanisms underlying the process are still incompletely understood (for example, it is still not clear whether the charged species transferred between the surfaces is an electron, ion, or microscopic quantities of material).^2–6^ Furthermore, experiments have shown that only insulating materials separate a significant amount of charge through contact electrification. Because the materials involved are insulators, the charge generated remains at the localized spot on the solid surface after contact; thus, it is difficult to transport the charge elsewhere for powering devices. In addition, since the phenomenon requires contact of two materials, an external driving force is typically needed to separate the charge. Despite these challenges, Wang and co-workers have made advances in this field by using a combination of contact electrification, electrostatic induction, and fabrication techniques at the micro- to nano-scale.^7,8^

When the interface involves a solid and a liquid, power can be generated in the form of streaming currents: by pumping an aqueous solution through a small (micro- to nano-sized) channel, a potential difference can be produced between the inlet and outlet of the channel.^9,10^ This phenomenon is mainly driven by the separation of charge at the interface between the fluid and the walls of the channel. This method of generating power (up to 240 pW per channel in a nano-sized channel^10^), however, requires pressure to drive the liquid through the small channels. It has been shown that this method can generate electricity by pumping liquid across solid surfaces of many different types of materials, such as carbon nanotubes^11^ and graphene.^12^ Power can also be generated through electrostatic contact and induction by pressing poly(dimethylsiloxane) (PDMS) periodically against a layer of water using a motor (or, potentially, from wave energy).^13^ Other methods include electrostatic induction of an existing charge in water (*i.e.*, the water is charged before it enters the system), or an existing charge on a solid surface (after an initial contact with water).^14^

In this study, we explored the possibility of harvesting the gravitational energy of water by separation of charge at the solid–liquid interface. More specifically, we seek to answer the question: can gravitational energy of water *alone* (*i.e.*, without the need to supply other types of energy, such as mechanical energy) generate a significant amount of power through contact electrification? Our rationale behind this study is that the gravitational energy of water is a proven source of energy that can produce a large quantity of power (*e.g.*, by hydroelectricity); hence, it may also be possible to produce a significant amount of power through contact electrification. The attractiveness of harvesting gravitational energy of water is obvious: this source of energy is clean, renewable, and is available in abundance naturally, such as in rivers, and rain. In addition, we believe that using water to separate charge at the water–solid interface overcomes the typical barrier in harvesting energy from contact electrification involving two solid surfaces. For contact between two solids, there is typically a need to incorporate additional features in the system to transfer and collect charges generated at localized spots on the insulating surfaces. Water, however, can flow naturally into a specific location (*e.g.*, a container) under its own weight, thus allowing the charge to be collected in a centralized spot. Another consideration in the design of our experiment is the ease and the cost of producing this power. We used only materials that are commonly available, and can be used immediately (*i.e.*, without the need for further modification).

**Fig. 1:**
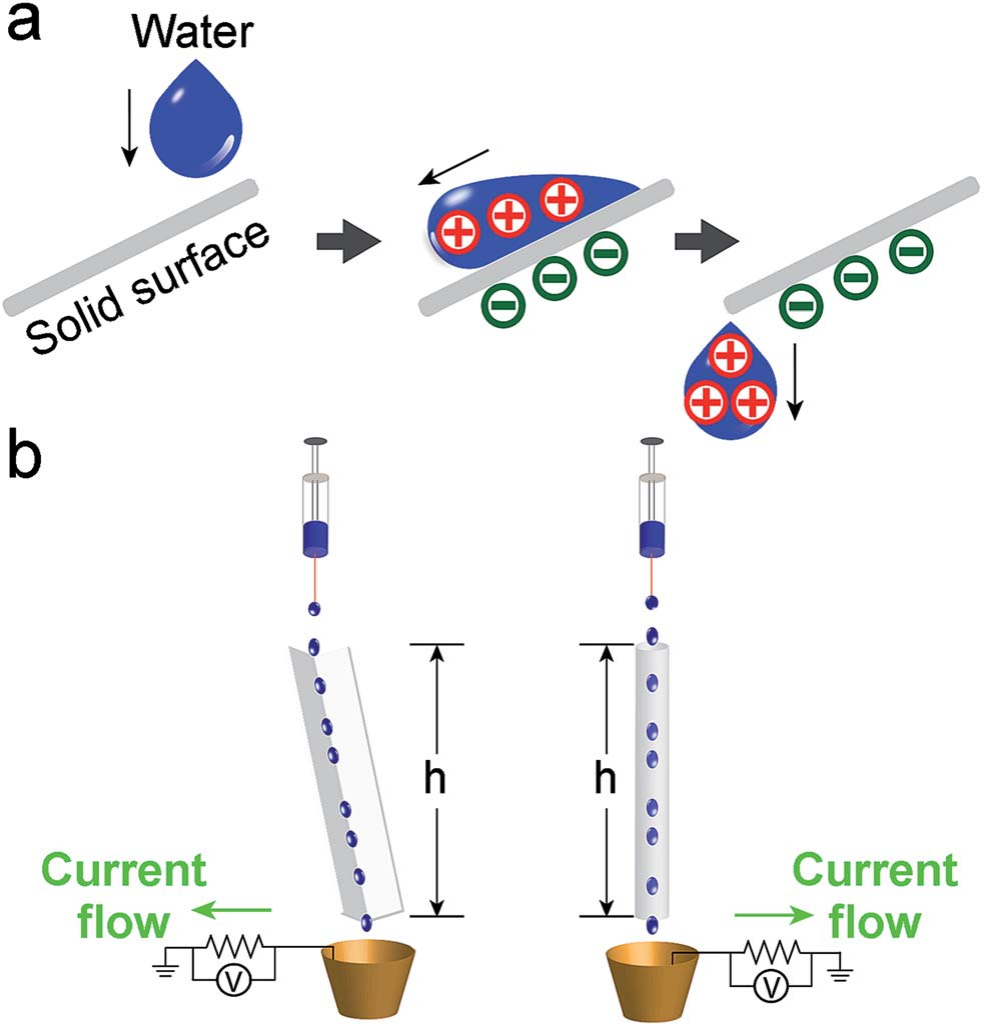
(a) When an initially uncharged drop of water flows down a solid surface due to gravity, charge separates at the interface. After falling off the surface, the water droplet acquires a net charge. It is thus possible to harvest the gravitational energy of water droplets for generating power. (b) The experimental setup for generating power involves either a L-shaped channel (scheme on the left), or a tube (scheme on the right).

Experimentally, we found that when water (initially uncharged) flows across, and then falls off a solid surface under its own weight, it acquires a net positive charge (Fig. 1a). Since the system involves the solid–liquid interface for developing electrification, we refer to the system as SLIDE. Our results show that it is possible to produce a power of up to ∼170 μW per tube continuously after optimizing the various factors influencing the system. Subsequently, we examined the movement of positive and negative charges in SLIDE. We also compared the amount of charge that can be produced by SLIDE with the amount that can be generated by a high-voltage power supply.

## Results and discussion

In our experiments, we used deionized water unless otherwise stated. Water was pumped at a regulated flowrate continuously onto a solid surface (Fig. 1b). (It is also possible to supply water from a natural source.) Two types of solid surfaces were used: L-shaped channels, or cylindrical tubes. After flowing water across the solid surface, we measured its charge by collecting it in a Faraday cup connected to an electrometer (model 6514, Keithley) (as discussed in a previous study;^4^ see ESI,† Section 1 for more details on the experimental methods and materials used). We also determined the power output of SLIDE by measuring the potential difference across a resistor connected to the Faraday cup and to the ground. By collecting the data using a computer, we could determine the power generated with respect to time. All experiments were conducted at room temperature and at a humidity of ∼70%. Since we are harvesting the gravitational energy of water, we can define an efficiency, *ε*_ff_, as the amount of energy generated by SLIDE (*i.e.*, the power generated integrated with respect to the duration of the experiment), *E*_S_, over the gravitational energy, *E*_g_, that the water loses. *E*_g_ is calculated by considering the total mass of water, *m*, that falls down the vertical height of the solid surface, *h* (see Fig. 1). The mathematical expression for the efficiency is shown in eqn (1), where *V* is the potential difference measured experimentally across a resistor of resistance *R*, *t* is time, *t*_f_ is the duration of the experiment, and *g* is the constant of acceleration due to gravity.1
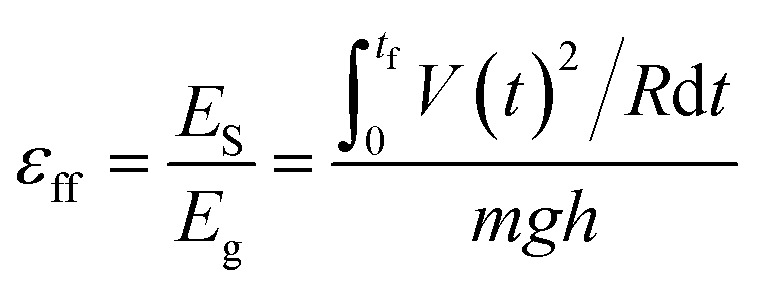



**Fig. 2:**
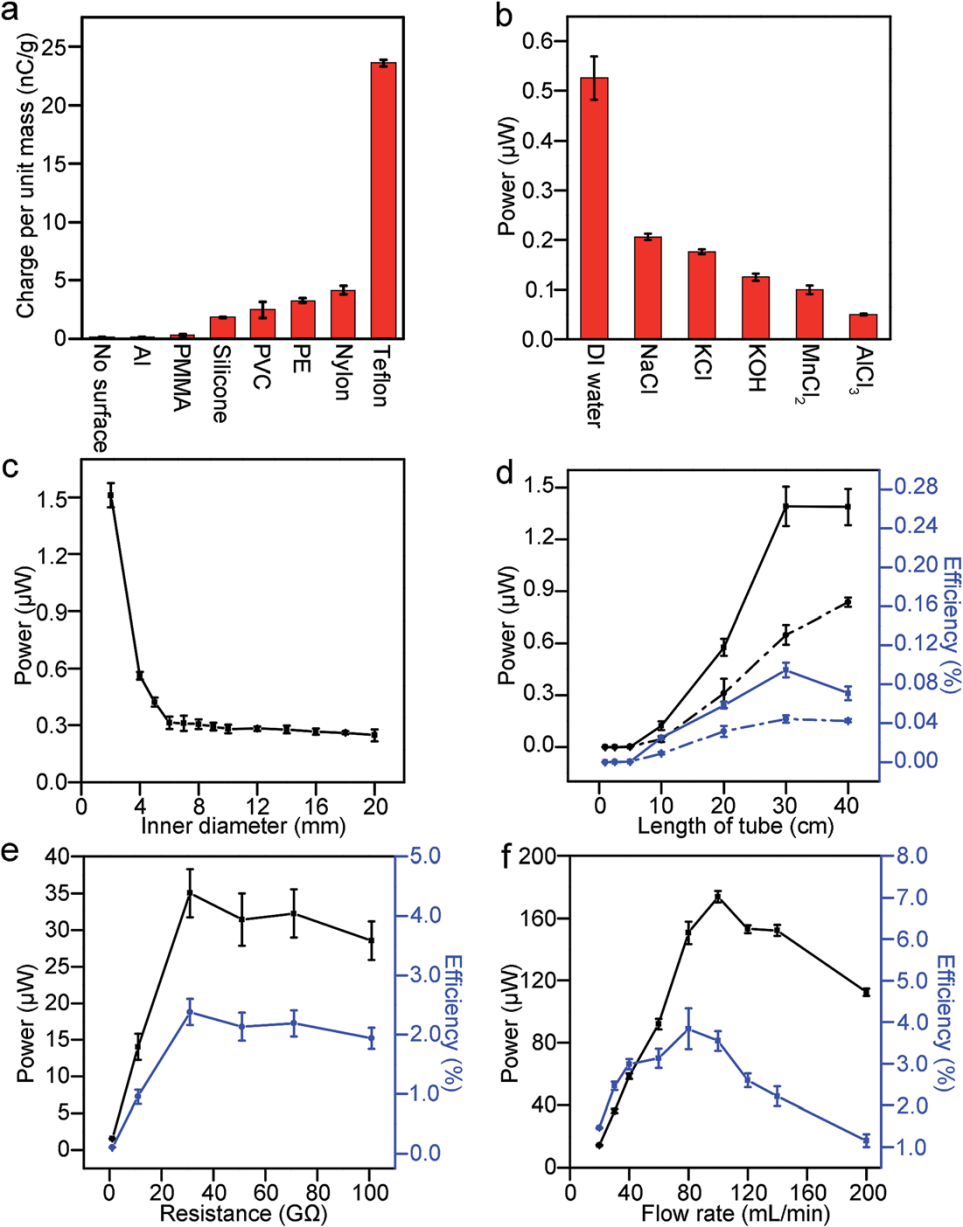
(a) Deionized (DI) water at a flowrate of 1 mL min^–1^ was flowed across L-shaped channels (length of 30 cm; tilted 10° to the vertical) of different materials. It acquired the most charge when it contacted a Teflon surface compared with other materials investigated (Al – aluminum, PMMA – poly(methyl methacrylate), PVC – poly(vinyl chloride), PE – polyethylene). Resistance used was 1 GΩ. (b) DI water generated the most power compared with water added with different types of salts. The solution was flowed down a Teflon tube (inner diameter of 4 mm; length of 30 cm) at a flowrate of 30 mL min^–1^. The resistance used was 1 GΩ. The concentration of all the salt solutions was 0.01 M. (c) The smaller the inner diameter of the tube used, the higher the power generated. Teflon tubes (30 cm in length) of different diameters were used. The flowrate was 30 mL min^–1^. The resistance was 1 GΩ. (d) The longer the tube, the higher the power generated (black line); however, the efficiency (blue line) shows a maximum at a length of 30 cm. Teflon tubes with an inner diameter of 2 mm (solid lines) and 4 mm (dotted lines) were used. The flowrate was 30 mL min^–1^. The resistance was 1 GΩ. (e) Both the power and efficiency reached a maximum when the resistance was 31 GΩ. DI water was flowed down a Teflon tube (inner diameter of 2 mm; length of 30 cm) at a flowrate of 30 mL min^–1^. (f) A maximum power of ∼170 μW and an efficiency of ∼3–4% were achieved when DI water flowed down a Teflon tube (inner diameter of 2 mm; length of 30 cm) at a flowrate of ∼80–100 mL min^–1^. Resistance used was 31 GΩ.

Whenever water interacts with a solid surface and is collected in the Faraday cup, a significant amount of potential difference is measured across the resistor; hence, power is generated. Importantly, we found that SLIDE does provide a continuous supply of power as long as there is a continuous supply of water (*e.g.*, for hours in our experiments). We investigated the amount of charge produced in the water droplets (radius ∼2 mm) with respect to the different types of materials used as the solid surface (see ESI,† Section 2 for the plots of charge generated *versus* zeta potential and contact angle of the different types of solid materials used). Fig. 2a shows that when the droplets fell directly into the Faraday cup (without contact with any solid surface), the amount of charge measured was negligible compared to the case when the droplets fell onto a non-metallic surface. This result shows that a solid surface is important in generating charged water droplets. The water droplets are charged positively for all the types of materials investigated. It is well known that charge can separate at the interface of solid and liquid.^15^ When the contact involves water and a nonionic material, Whitesides and co-worker proposed that a negatively-charged species in water (specifically, the hydroxide ions) may adsorb preferentially onto the solid surface.^3^ After the water moves away from the solid surface, the kinetic energy of water separates the charge permanently, thus leaving the solid surface with the negatively-charged species and a net positive charge in water.

Since the Teflon surface produced the most charge out of the different common materials we investigated, we used only Teflon for subsequent experiments. This observation is in accordance with the triboelectric series (an empirically established list of materials that ranks the extent by which each material charges positively or negatively with other materials). With respect to the liquid, we added different types of salts (monovalent or multivalent) to the deionized water; it was found that pure deionized water produced the most charge (Fig. 2b). Interestingly, although salt reduces the amount of energy produced in this system, it is actually an important component for the harvesting of energy in other systems. For example, it has been reported that power can be generated *via* a concentration gradient of salt across a nanopore.^16,17^ In this case, the salt solution is necessary for supplying the cations and anions, which are then separated by a pre-existing charge on the surface of the nanopore. The system described in this study, however, depends on the separation of charge at the solid–liquid interface, and not on a pre-existing charge on the surface of the solid. Our result suggests that any salt added to the liquid may give rise to a condition such that it is energetically more favorable for the negatively-charged species to remain in the liquid than to be transferred onto the solid surface (see ESI,† Section 3, for a fuller discussion of the effect of electrolytes on the separation of charge). Since contact electrification is a nonequilibrium phenomenon, it may be possible for the mobility of ions to influence the amount of charge separated. Our experiment shows that conductivity (which is proportional to the mobility of ions) is negatively correlated with the power generated (see ESI,† Section 3, for a plot of conductivity *versus* power).

Subsequently, we explored ways to maximize the power and the efficiency, *ε*_ff_, of SLIDE through optimizing the many factors that can influence the system. After investigating the types of materials used, we examined the geometry of the solid surface needed. First, we found that the amount of charge generated in a Teflon tube was significantly higher than a L-shaped Teflon surface (to be discussed in Fig. 4). For a Teflon tube, we investigated the influence of the inner diameter and the length of the tube on the power generated. Results show that the smaller the inner diameter of the tube, the higher the power generated (Fig. 2c). We believe that this result is a consequence of a larger area of contact between the water and the tube. We measured experimentally the area of contact of water droplets in tubes of different diameter, and found that the area of contact is larger for smaller tubes (see ESI,† Section 4, for more detail). If a larger area of contact separates more charge, then a droplet with a smaller radius should also acquire a higher charge per unit mass. Experiments (see ESI,† Section 5) show that smaller droplets do result in a larger charge per unit mass. We also performed the same experiment with a tube smaller than 2 mm (*i.e.*, a tube with an inner diameter of 1 mm), but the water overflowed from the top at the flowrate of 30 mL min^–1^. With respect to the length of the tube, the power increased with increasing length for up to ∼30 cm; a length longer than ∼30 cm did not produce significantly more power (Fig. 2d). The efficiency, on the other hand, showed a peak at 30 cm. The decrease in efficiency for longer tubes is due to the increase in the input of gravitational energy (as a consequence of a higher height, *h*). Therefore, we chose to work with a Teflon tube with an inner diameter of 2 mm, and a length of 30 cm.

We then varied the resistance and found that maximum power and efficiency are obtained when the resistance is 31 GΩ (Fig. 2e). Using this resistance, we examined different flowrates of water flowing down the tube (Fig. 2f). Results show that a power of up to ∼170 μW (time-averaged) and an efficiency, *ε*_ff_, of up to ∼3–4% are obtained at a flowrate of ∼80–100 mL min^–1^. A flowrate of 100 mL min^–1^ in a tube with an inner diameter of 2 mm means that the velocity of the water flowing down the tube is 0.5 m s^–1^—it is possible for raindrops to flow down the tube at this velocity since the terminal velocity of raindrops (*e.g.*, with a diameter of 2 mm) is known to be around an order of magnitude higher than 0.5 m s^–1^.^18^ Section 6 in the ESI† gives a more detailed discussion of the method of calculating the efficiency at these conditions.

**Fig. 3:**
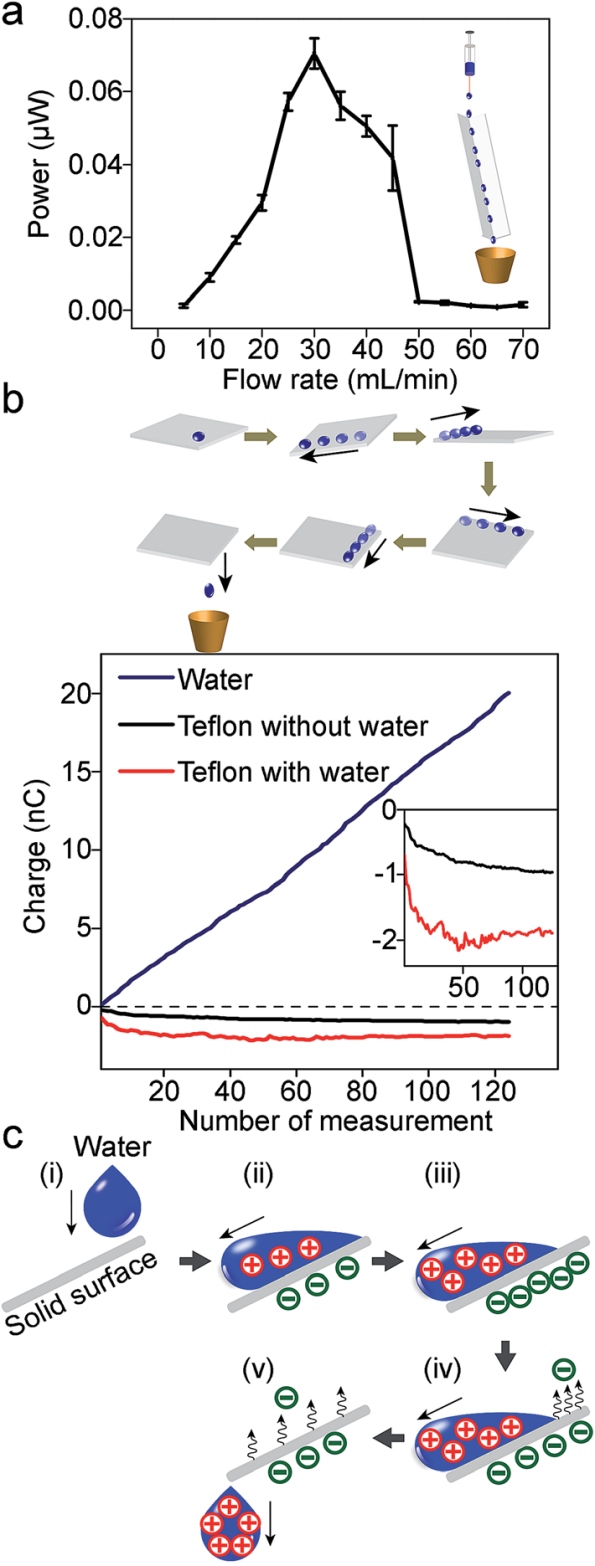
(a) The plot of power against different flowrates of DI water flowing down a Teflon L-shaped channel (30 cm; tilted at 10° to the vertical). A distinct peak is observed at moderate flowrates (∼30 mL min^–1^). Resistance was 1 GΩ. This trend is similar to water flowing down a Teflon tube (Fig. 2f). (b) In order to investigate the movement of charges, a water droplet was placed on a slab of Teflon (1 cm × 1 cm × 1 mm); the slab was rotated such that the droplet moved across its four edges and corners. After that, the charges of the droplet and the slab were measured using a Faraday cup connected to an electrometer. The plot at the bottom shows the measurements of the charge for the droplets (blue line), and the slab of Teflon (red line). The experiment was repeated for more than a hundred droplets; the *x*-axis indicates the number of times repeated. The results show that the droplets are constantly charged positively (as indicated by the approximately constant gradient of the blue line); however, the charge of the slab (red line) reached saturation when in contact with ∼20 droplets. As a control experiment, the charge of a slab of Teflon not in contact with water was also measured (black line). The inset shows the enlarged plots of the charge of the slab with and without contacting the water droplets. (c) Illustration of the movement of positive and negative charges when a water droplet moves across a solid surface.

Unexpectedly, Fig. 2f shows that power decreases when the flowrate increases beyond ∼100 mL min^–1^ for the case when the resistance is 31 GΩ. This result is unexpected because, intuitively, one would assume that when there is more water—and hence, more gravitational energy—SLIDE would produce more electrical energy. This observation appears to be universal across various experimental conditions. We repeated the experiment in Fig. 2f but with a resistance of 1 GΩ (see ESI,† Section 7); the trend is observed to be similar to Fig. 2f. When we repeated the experiment using a L-shaped Teflon channel, we again observed that the power reached a maximum at moderate flow rates (this time at ∼30 mL min^–1^), and then decreased with increasing flow rate (Fig. 3a). The advantage of using a L-shaped Teflon channel is that it allows us to inspect visually the pattern of flow of water across the channel more easily. We observed that water remained as individual water droplets for low flow rates (*i.e.*, <30 mL min^–1^), while a continuous stream of water was observed at high flow rates (*i.e.*, >50 mL min^–1^). At moderate flow rates (*i.e.*, >30 mL min^–1^ and <50 mL min^–1^), we observed that the flow switched intermittently between droplets and a continuous stream; this irregular pattern of flow was likely due to the slightly inconsistent rate of flow due to the peristaltic pump we used. This observation suggests that discrete water droplets are capable of generating much larger power than a continuous stream of water.

In addition, a second point needs to be clarified: if a continuous stream of water is constantly charged positively, then what happens to the negative charge that is generated at an equal rate? One argument is that the solid surface accumulates negative charge constantly; after some time, the surface may be saturated with negative charge such that it can no longer produce positively-charged water. On the other hand, we observed experimentally that water can be charged positively for at least a few hours.

In order to understand the two points (stated, respectively, in the previous two paragraphs) better, we investigated the movement of positive and negative charges in the system. Specifically, we measured and monitored the charges generated on both the water droplet and the solid surface. A water droplet was first brought into contact with a small slab of Teflon (1 cm × 1 cm × 1 mm). The solid surface was then gradually rotated such that the drop of water moved across the four edges and corners of the slab (see scheme in Fig. 3b). All manipulations were done using polybutylene terephthalate tweezers; these tweezers were found previously to have insignificant influence over the charge of the materials they contacted.^4^ After rotating the surface, we measured the charge of the water droplet and the slab of Teflon separately. We repeated the experiment for more than a hundred water droplets. Results show that while each water droplet charges to similar extents (as indicated by the approximately constant gradient of the blue line in Fig. 3b), the charge of the slab of Teflon saturates after contact with around twenty droplets. Eventually, the total amount of positive charge accumulated by all the water droplets is ∼20 nC, while the slab of Teflon has only a maximum negative charge of ∼2 nC. We repeated this experiment five times, and obtained similar results (the plot in Fig. 3b shows the results of one representative experiment).

The law of conservation of charge, however, states that there should be an equal amount of positive and negative charges generated in the process. In order to explain the unequal amounts of positive and negative charges, we propose that the slab of Teflon discharges by transferring its negative charge to gaseous molecules in the atmosphere. This phenomenon may be similar to the results found in previous studies;^4,19,20^ when charge accumulates on the solid surface, the electric field generated may eventually exceed the dielectric breakdown strength of the atmosphere. Under this circumstance, gaseous molecules ionize. The positive gaseous ions are attracted to the solid surface (and deposit on it), while the negative ions are repelled away from the surface.

As an order-of-magnitude analysis, we assume that the electric field is homogeneous across the surface of the slab of Teflon (this assumption is more accurate for regions away from the edges of the slab). In this case, we can apply Gauss's law to obtain a simple relationship between the electric field, *E*, the charge density, *σ*, and permittivity, *ε* (eqn (2)).2
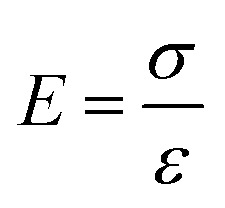



Since the charge on the slab of Teflon is ∼2 nC, the charge density (for a surface area of 1 cm^2^), *σ*, is ∼20 μC m^–2^. Accordingly, the electric field is calculated to be 2.3 MV m^–1^. This value is close to the dielectric breakdown strength of the atmosphere, which is^21^ ∼3 MV m^–1^. Thus, it is possible for the slab of Teflon to discharge by ionizing gaseous molecules, and transfer its charge to the surrounding atmosphere. In short, charge is conserved in the system when water droplets gain positive charge continuously (with a constant supply of water), while the solid surface transfers negative charge continuously to its surrounding atmosphere (Fig. 3c).

Following this discussion, an explanation for the drastically lower power measured for higher flow rates of water as compared to that of lower flow rates is as follows (see, for example, Fig. 3a). At high flow rates, a continuous stream of water covers the surface of the slab of Teflon; this coverage prevents the surface from interacting with the atmosphere. At low flow rates, water flows through the solid surface as discrete droplets. The spaces between the droplets allow the solid surface to discharge whenever the electric field exceeds the dielectric breakdown strength of the atmosphere.

**Fig. 4:**
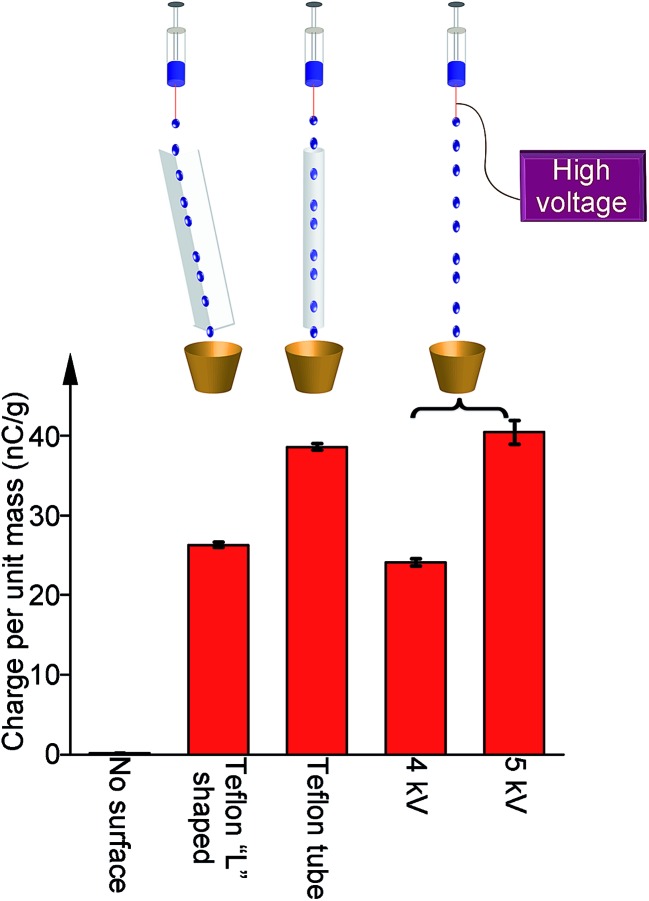
An electrical potential of ∼4–5 kV from a power supply was required to generate the amount of charge in a water droplet similar to that generated by contacting the droplet with either a L-shaped channel or a tube. When water droplets were not in contact with any solid surface, they did not contain any charge.

Besides optimizing the amount of power produced, we also compared the amount of charge generated in SLIDE *versus* the amount generated using a high-voltage power supply. For the latter, we connected the metallic needle of a syringe to a high-voltage power supply (model 610C, TREK). We applied a specific amount of electrical potential to the needle, and pumped water droplets out of the syringe. After collecting the water droplets in a Faraday cup, we measured the amount of charge generated in the droplets. We found that the amount of charge (per unit mass) generated by SLIDE is equivalent to that produced by a voltage of ∼4–5 kV (*i.e.*, ∼4 kV for a L-shaped channel, and ∼5 kV for a tube) using an external power supply (Fig. 4).

## Conclusions

**Fig. 5:**
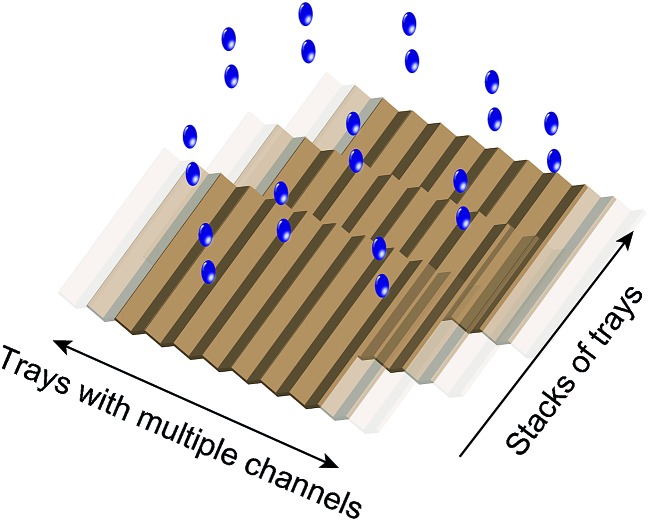
Multiple L-shaped channels can be connected to form a two-dimensional tray; a number of these two-dimensional trays can be stacked in the third dimension. It is proposed to install this structure in regions of abundant rainfall, or in the paths of flowing water in order to harvest the gravitational energy of water.

Experimentally, we found that when water flows down a solid surface under its own weight, it gains a net positive charge. We showed that it is possible to generate power using this system: a continuous flow of water droplets (∼100 mL min^–1^) generates a continuous supply of power of ∼170 μW with a single Teflon tube of inner diameter 2 mm. The efficiency of SLIDE (*i.e.*, energy generated over the gravitational energy that the water loses) can reach up to ∼3–4%. Importantly, this phenomenon relies purely on the gravitational energy of water to supply power—sources of gravitational energy of water are found in abundance in nature, such as in rain, and streams of water. This energy is renewable, clean, and “freely” available. SLIDE is technically simple and inexpensive to operate: it uses only commonly-available materials (*e.g.*, Teflon). It can be scaled up easily. As an illustration, one way of scaling up SLIDE is to build multiple L-shaped channels connected together to form a two-dimensional tray. Multiple such trays can also be stacked in the third dimension (Fig. 5). If a single L-shaped channel is 1 cm wide, a two-dimensional tray of 5 meters wide has 500 channels. If we stack 20 trays in the third dimension, the power generated from this solid structure can be amplified (from a single L-shaped channel) by a factor of 10^4^. If we multiply this factor with the power generated by SLIDE under ideal conditions, this structure can potentially generate a power on the order of ∼1 W. In addition, we found experimentally that the water can be reused repeatedly to generated energy, thus it may be possible to operate SLIDE in multiple sequential stages. It is possible for a structure of this dimension to be placed in common areas, such as on rooftops. SLIDE also has the advantage that it converts gravitational energy directly into electrical power. This consequence of SLIDE is different from, for example, hydroelectric power; hydroelectric power first converts gravitational energy into mechanical energy (*i.e.*, by turning a turbine), and then converts the mechanical energy into electrical energy (by using a separate system that enables this conversion). SLIDE does not require any other additional sources of energy to be supplied into the system (as opposed to methods such as the use of streaming current which requires pressure to drive liquid through micro- or nano-sized channels).

We showed that SLIDE is capable of producing charged water droplets that would otherwise require the use of a high-voltage power supply. Experimentally, we found that a high voltage of ∼5 kV provided by a power supply is needed to produce a droplet with similar amount of charge as a droplet flowing down a Teflon tube. Thus, SLIDE may be an inexpensive way to produce charged water droplets for purposes involving high electrical potential (*e.g.*, for applications related to printing, or electrospraying^22,23^). In terms of charge balance, our results suggest that while a continuous stream of water is charged positively, an equal amount of negative charge is transferring constantly to the surroundings; thus, SLIDE can produce power as long as there is a supply of water.

Currently, the amount of power generated by SLIDE is still much smaller than other means of power (*e.g.*, hydroelectricity), even when it is operating at ideal conditions. There is thus a need to further investigate and modify the system (*e.g.*, through using different materials, coatings, and/or varying the topology of the surface of the solid) in order to increase the amount of power generated.
