# Supplementary material for: Using the gravitational energy of water to generate power by separation of charge at interfaces
Source: Chem Sci. 2015 Mar 26;6(6):3347–53. doi: 10.1039/c5sc00473j (PMC5490415; doi:10.1039/c5sc00473j)
Supplement: Supplementary file 1 [file SC-006-C5SC00473J-s001.pdf]

Electronic Supplementary Material (ESI) for Chemical Science.
This journal is © The Royal Society of Chemistry 2015

Supporting Information

Using Gravitational Energy of Water to Generate Power by Separation of Charge at Interfaces

Yajuan Sun, Xu Huang, and Siowling Soh*

Department of Chemical and Biomolecular Engineering, National University of Singapore, 4 Engineering Drive 4, Singapore 117585, Singapore

* To whom correspondence may be addressed: chessl@nus.edu.sg

1. Materials and Methods

We used deionized water (Cartridge type MC: DS, MicroMeg) rather than other types of water (e.g., tap water) so that the results could be reproducible and not subjected to other sources of uncertainty. In order to deliver a regulated amount of water onto the solid surface, we pumped water using either a syringe pump (Legato 210, KD Scientific) for small flow rates, or a peristaltic pump (Masterflex) for high flow rates. Usually, water was pumped onto either one of the two types of solid surfaces: a L-shaped channel, or a tube. For a L-shaped channel, we used either aluminum, nylon, poly(methyl methacrylate) (PMMA), polyethylene (PE), poly(vinyl chloride) (PVC), silicone, and polytetrafluoroethylene (PTFE, or Teflon®). Note that the ® symbol is omitted in the text for simplicity. The tubes of different diameters are made of Teflon, a perfluoroalkoxy copolymer (PFA). Charge was measured by a home-made Faraday Cup connected to an electrometer (model 6514, Keithley) (as discussed in a previous study[1]). Briefly, the Faraday Cup measures the charge of any objects placed within it by inducing a charge on the inner metallic surface of the Cup.

We also determined the power output of SLIDE by connecting the Faraday Cup to a resistor (or, a series of resistors), and then to ground. By measuring the potential difference across the resistor with the electrometer, we determined the power generated by SLIDE. The values for the potential difference throughout the whole duration of the experiment were recorded by a computer through the use of a program that served as an interface between the electrometer and the computer (i.e., LabVIEW, from National Instruments).

2. Charge per Unit Mass Generated Versus Zeta Potential and Contact Angle

In order to investigate if there is any correlation between the charge generated by SLIDE and the zeta potential and the contact angle of the different types of solid materials in contact with water, we have plotted the graph as shown in ESI Fig. S1. In general, the amount of charge generated does not seem to have any dependence on zeta potential or contact angle. This result is not surprising since it is known that the phenomenon of contact electrification is likely to involve multiple fundamental mechanisms, and may not correlate with a single material property.

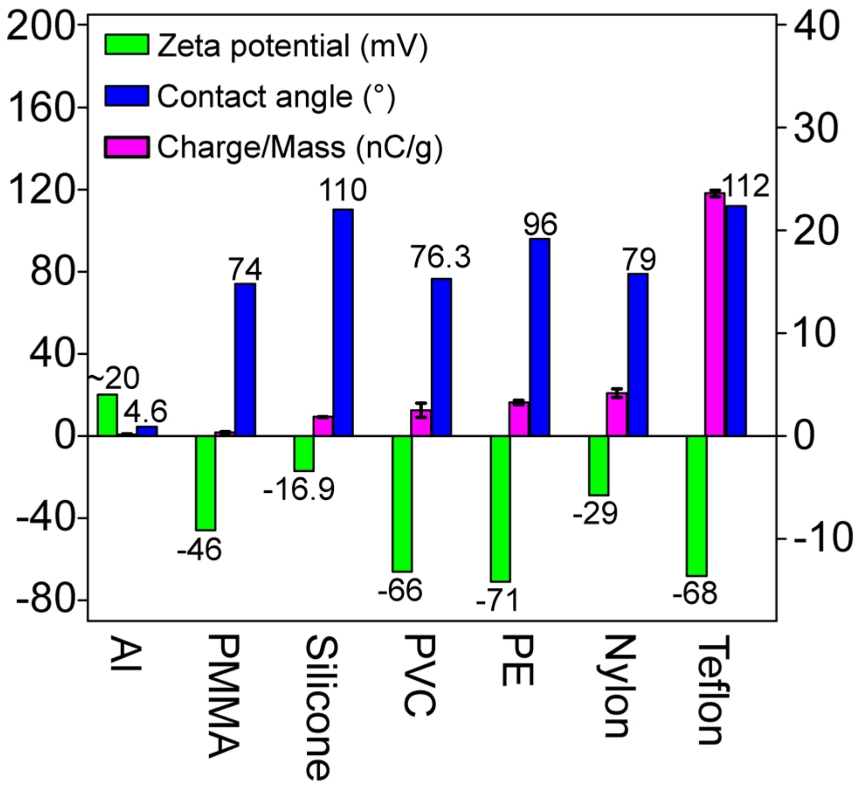

Fig. S1 Plot of charge generated versus zeta potential and contact angle. The *y*-axis on the left represents values for zeta potential and contact angle. The *y*-axis on the right represents values for charge per unit mass generated. Data for zeta potential and contact angle are obtained from these references.[2-10]

3. Effects of Electrolytes on Charge Separation

In our experiments, we found that when electrolytes were added into deionized water, the amount of power generated decreased. This result may be a consequence of a lesser amount of charge separated at the interface of solid and liquid when the liquid contains electrolytes. An explanation is as follows.

For the case when water (i.e., without any electrolyte) comes into contact with a solid surface, it has been proposed previously that a negatively-charged species in water (specifically, hydroxide ions) tends to adsorb preferentially onto the solid surface. When the surfaces separate, the kinetic energy of the water separates the ions permanently, thus leaving the negatively-charged species on the solid surface, and a net positive charge in water. The amount of charge separated, therefore, depends on whether is it more energetically favorable for the negatively-charged species to stay on the solid surface or the liquid phase. Our results shows that when electrolytes are added to water, the amount of power generated decreased; this observation suggests that it is energetically more favorable for the negatively-charged species to remain in the liquid phase when it contains electrolytes than when it does not. One explanation for this observation is that the positively-charged ions in the solution may gather closer to the interface when the solution contains more electrolytes (i.e., the debye length is known to be smaller for a solution with a greater ionic strength); the proximity of the positively-charged ions at the interface may increase the tendency for the negatively-charged ions to remain in the liquid phase.

On the other hand, ionic strength is clearly not the only factor influencing the amount of charge separated (if it were the only factor, the monovalent salt solutions of the same concentration would separate the same amount of charge; however, the power generated is different for NaCl, KCl, KOH, and HCl). Because contact electrification is a nonequilibrium

phenomenon, it may be possible that the *rates* at which the ions move may also influence the amount of charge separated. An easily measurable quantity is conductivity — it is dependent on both the ionic strength and the mobility of the ions. We measured the conductivity of the different salt solutions using a conductivity meter (Thermo Scientific), and plotted the conductivity of several salt solutions against the power generated by SLIDE (ESI Fig. S2). The plot shows that, in general, the greater the conductivity, the lesser the power produced. This trend suggests that the larger the mobility of the ions, the higher the tendency for the negatively-charged ions to remain in the liquid phase. As an example, if we consider KCl and KOH solutions, we observed that the power generated by KCl (conductivity, 0.16 S/m) is 0.18 W while that generated by KOH (conductivity, 0.27 S/m) is 0.13 W. From literature, we know that the mobility of Cl^- ions is 7.9×10^{-4} cm^2 sec^{-1} V^{-1}, and the mobility of OH^- ions is at a higher value of 2.1×10^{-3} cm^2 sec^{-1} V^{-1}.

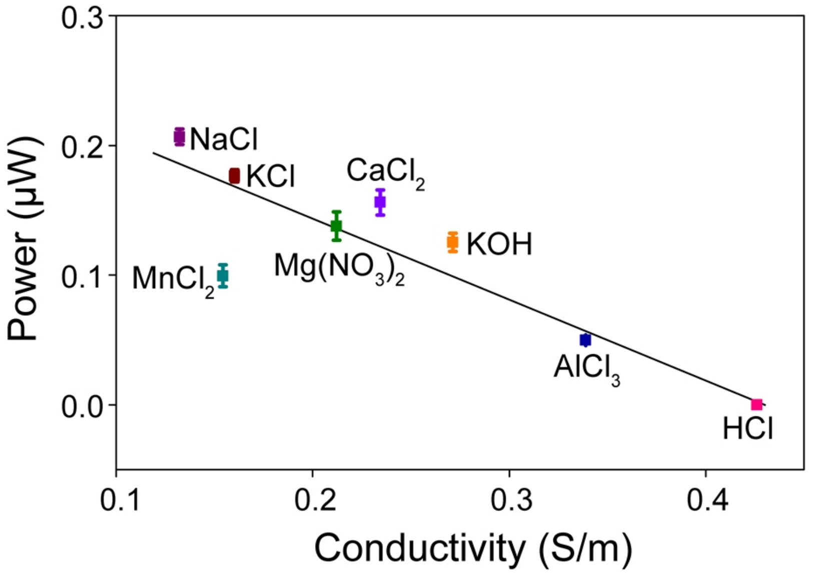

Fig. S2 Power generated with respect to the conductivity of the liquid. The concentration of all the salt solutions is the same at 0.01 M.

4. Measuring Area of Contact of a Water Droplet Resting in a Tube

In the main text, we discussed the influence of the area of contact between the water droplet and the Teflon tube over the charge generated on the droplet (see Fig. 2c in the main text). By using tubes of different diameters, we were able to vary the contact area between the water droplet and the tube.

We determined the contact area as follows. First, we placed a water droplet (~ 0.03 g) within the tube (ESI Fig. S3a). Since the tube was translucent, we were able to image the water droplet within the tube. A camera was placed at the bottom of the tube, and an image was taken upward in order to capture the contact area. After taking the image, we traced the boundary of the water droplet using an image processing software (ImageJ, http://imagej.nih.gov/ij/). After obtaining a series of points (i.e., the points with the x and y coordinates of the boundary), we integrated numerically (Matlab 2013a, The MathWorks, Inc) the boundary to obtain the contact area. In the process of integration, we took into account of the curvature of the tube; the curved contact area can be calculated by knowing the inner diameter of the tube. Fig. S3b shows the relationship between the inner diameter of the tube and the contact area determined by this method.

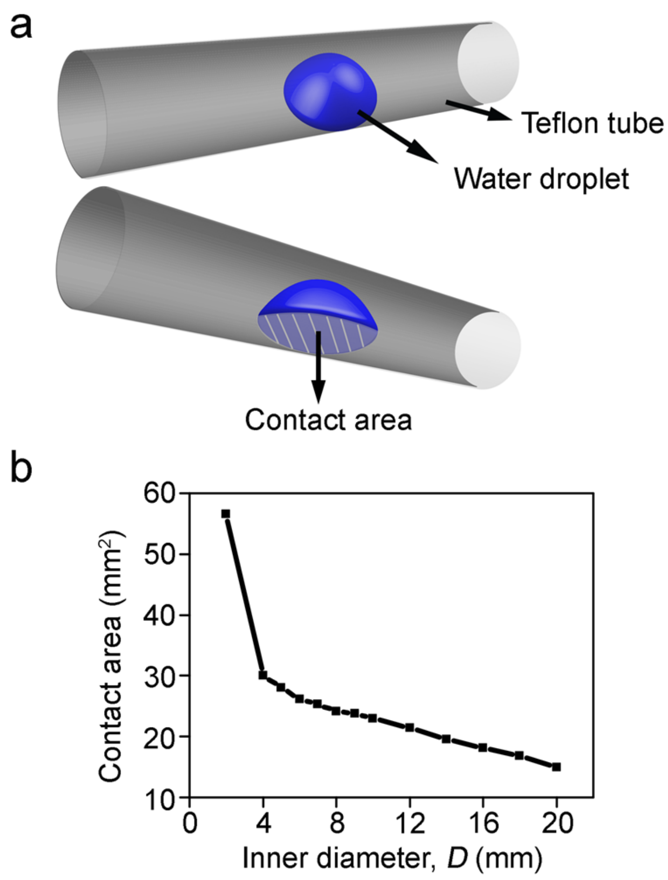

Fig. S3 (a) By placing a water droplet in a tube (placed horizontally), the area of contact between the droplet and the tube can be imaged. Subsequently, the image can be analyzed and the actual area of contact can be calculated numerically. (b) Plot of the contact area with the inner diameter of the tube.

5. Water Droplets with Smaller Radii Gained Higher Charges per Unit Mass

An experiment was performed where water droplets were pumped out of syringes with different diameters of the syringe needles; hence droplets of different diameters were obtained. The droplets flowed across a Teflon tube (30 cm; inner diameter of 4 mm), and their charges were measured. Results show that the smaller the droplet, the higher the charge per unit mass generated. This observation suggests that a greater amount of charge is separated per unit mass when the area of contact between water and the solid surface is larger.

A second explanation for this trend is that since the dielectric breakdown strength of the atmosphere is constant, the charge per unit mass should theoretically be inversely proportional to the size of the droplets. As a basis of analysis, we first approximate the geometry of the water droplet after it leaves the Teflon tube as a sphere. In this case, we can use Coulomb's law to estimate the electric field produced by the droplet as shown in Equation S1, where *E* is the electric field, *q* is the charge, *ε* is the permittivity, and *r* is the radius.

$$E = \frac{q}{4\pi\varepsilon_0 r^2} \tag{S1}$$

Since the radius of the droplets are ~ 2 mm, its mass is ~ 0.034 g. The charge of the droplet, *q*, according to ESI Fig. S4 (~ 35 nC/g) is ~ 1.2 nC. According to Equation S1, this charge gives an electric field of ~ 2.6 MV/m — this magnitude of the electric field is close to the dielectric breakdown strength of air, which is 3 MV/m. There is, therefore, the possibility that the water droplet is typically charged to the limit of dielectric breakdown of air. If we now assume that the electric field, *E*, is constant for droplets for all the sizes, we can re-arrange the terms in Equation S1 to get an inverse relationship between the charge per mass and the radius of the droplet, $q/m \sim 1/r$. Thus, a smaller droplet should give a higher charge per mass.

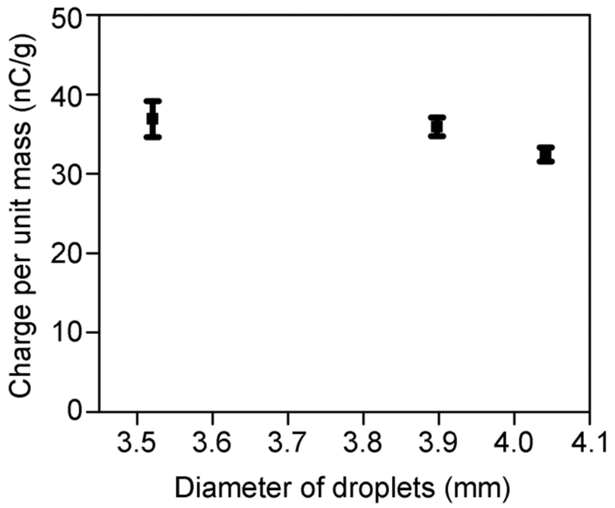

Fig. S4 Water droplets with smaller radii acquired higher charges per unit mass.

6. Method of Calculating Maximum Efficiency

We discussed in the main text that an efficiency of up to ~ 3 – 4 % can be achieved when DI water flows down a Teflon tube (inner diameter, 2 mm, and length, 30 cm) at a flowrate of ~ 80 mL/min – 100 mL/min (resistance, 31 GΩ). We defined the efficiency, ε_{ff}, as the amount of energy generated by SLIDE over the energy that is supplied to SLIDE — that is, the amount of gravitational energy that the water loses. An argument is that the energy supplied to SLIDE may include both the gravitational energy that the water loses and the kinetic energy of water as it flows out of the syringe pump at a flowrate of ~ 80 mL/min – 100 mL/min and enters the Teflon tube.

In our analyses, we have not considered the kinetic energy in the calculation of efficiency. The reason is as follows. In general, the energy balance for SLIDE includes the energy input to the system in the form of the gravitational energy and the kinetic energy of the

flow; the energy output includes the electrical energy produced and the kinetic energy that leaves the system (i.e., due to the water that flows out of the bottom end of the tube). Importantly, we consider the kinetic energy that leaves the system as a form of waste. If the condition is true that the velocity of water is the same throughout the length of the tube, the kinetic energy that enters SLIDE only contributes to the energy wasted, and does not contribute to the separation of charge; therefore, we did not include this kinetic energy in our analyses. Furthermore, we expect, theoretically, that the velocity should be similar throughout the tube at steady-state (except for the possibility that air pockets can be slightly compressed, if any, at the bottom of the tube); experimentally, we observed is that the velocity is visually similar throughout.

7. Power Increases and then Decreases with Increasing Flowrate of Water

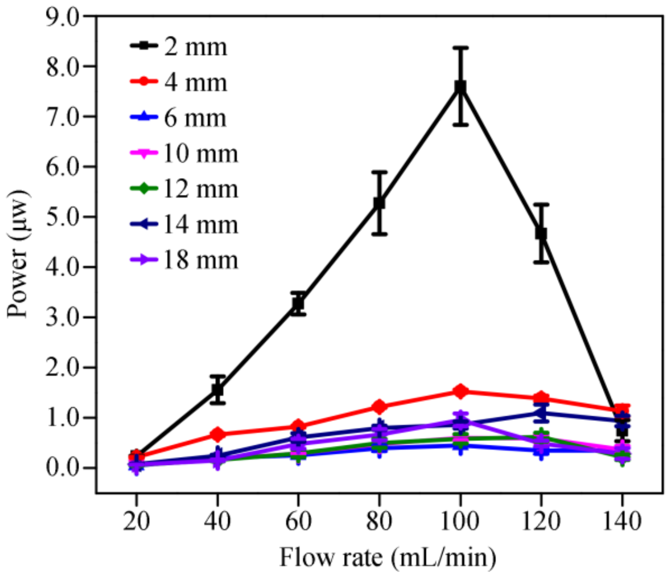

Fig. S5 In Fig. 2f of the main text, it is found that there is a distinct peak when power is plotted against the flowrate of water for the case when the resistance is 31 GΩ. Here, a similar trend is

observed for the case when resistance used is 1 GΩ. Maximum power is observed for various sizes of the Teflon tubes when the flowrate of water is approximately ~ 100 mL/min.

References:

1 S. Soh, S. W. Kwok, H. Liu and G. M. Whitesides, *J. Am. Chem. Soc.*, 2012, **134**, 20151-20159.

2 J. D. Bernardin, I. Mudawar, C. B. Walsh and E. I. Franses, *Int. J. Heat Mass Transfer*, 1997, **40**, 1017-1033.

3 J. Wu and N. Y. Lee, *Lab Chip*, 2014, **14**, 1564-1571.

4 M. T. Nazir, J. Xingliang and S. Akram, *Int. J. Electr. Comput. Sci.*, 2012, **12**,1-8.

5 P. Bouriat, P. Saulnier, P. Brochette, A. Graciaa and J. Lachaise, *J. Colloid Interface Sci.*, 1999, **209**, 445-448.

6 N. Kuehn, H. J. Jacobasch and K. Lunkenheimer, *Acta Polym.*, 1986, **37**, 394-396.

7 K. W. Powers, M. Palazuelos, S. C. Brown and S. M. Roberts, *Characterization of nanomaterials for toxicological evaluation*, John Wiley & Sons, United Kingdom, 2009.

8 L. S. McCarty and G. M. Whitesides, *Angew. Chem., Int. Ed.*, 2008, **47**, 2188-2207.

9 H. Busscher, G. Geertsema-Doornbusch and H. Van der Mei, *J. Biomed. Mater. Res.*, 1997, **34**, 201-209.

10 *US Pat.*, 10 569 919, 2003.
